# Natural Autoimmunity to Selenoprotein P Impairs Selenium Transport in Hashimoto’s Thyroiditis

**DOI:** 10.3390/ijms222313088

**Published:** 2021-12-03

**Authors:** Qian Sun, Sebastian Mehl, Kostja Renko, Petra Seemann, Christian L. Görlich, Julian Hackler, Waldemar B. Minich, George J. Kahaly, Lutz Schomburg

**Affiliations:** 1 Institute for Experimental Endocrinology, Charité-Universitätsmedizin Berlin, 13353 Berlin, Germany; qian.sun@charite.de (Q.S.); sebastian.mehl@mac.com (S.M.); Kostja.Renko@bfr.bund.de (K.R.); seemann@selenomed.com (P.S.); christian.goerlich@charite.de (C.L.G.); Julian.hackler@charite.de (J.H.); Waldemar.minich@charite.de (W.B.M.); 2 Freie Universität Berlin, Humboldt-Universität zu Berlin, Berlin Institute of Health, 13353 Berlin, Germany; 3 German Federal Institute for Risk Assessment, Department Experimental Toxicology and ZEBET, 12277 Berlin, Germany; 4 selenOmed GmbH, 10965 Berlin, Germany; 5 Johannes Gutenberg University Medical Center, Department of Medicine I, 55101 Mainz, Germany

**Keywords:** antioxidative defense, autoantibody, glutathione peroxidase, Hashimoto’s thyroiditis, trace element

## Abstract

The essential trace element selenium (Se) is needed for the biosynthesis of selenocysteine-containing selenoproteins, including the secreted enzyme glutathione peroxidase 3 (GPX3) and the Se-transporter selenoprotein P (SELENOP). Both are found in blood and thyroid colloid, where they serve protective functions. Serum SELENOP derives mainly from hepatocytes, whereas the kidney contributes most serum GPX3. Studies using transgenic mice indicated that renal GPX3 biosynthesis depends on Se supply by hepatic SELENOP, which is produced in protein variants with varying Se contents. Low Se status is an established risk factor for autoimmune thyroid disease, and thyroid autoimmunity generates novel autoantigens. We hypothesized that natural autoantibodies to SELENOP are prevalent in thyroid patients, impair Se transport, and negatively affect GPX3 biosynthesis. Using a newly established quantitative immunoassay, SELENOP autoantibodies were particularly prevalent in Hashimoto’s thyroiditis as compared with healthy control subjects (6.6% versus 0.3%). Serum samples rich in SELENOP autoantibodies displayed relatively high total Se and SELENOP concentrations in comparison with autoantibody-negative samples ([Se]; 85.3 vs. 77.1 µg/L, *p* = 0.0178, and [SELENOP]; 5.1 vs. 3.5 mg/L, *p* = 0.001), while GPX3 activity was low and correlated inversely to SELENOP autoantibody concentrations. In renal cells in culture, antibodies to SELENOP inhibited Se uptake. Our results indicate an impairment of SELENOP-dependent Se transport by natural SELENOP autoantibodies, suggesting that the characterization of health risk from Se deficiency may need to include autoimmunity to SELENOP as additional biomarker of Se status.

## 1. Introduction

Autoimmune thyroid disease (AITD) is characterized by an inappropriate interaction of immune cells with thyroid proteins. An activated immune system, inflammation, and lymphocytic infiltration into the thyroid gland, accompanied with autoantibodies (aAb) to thyroid antigens, are hallmarks of AITD [1]. Natural aAb levels are commonly determined in AITD as diagnostic markers, which support the diagnosis, correlate to disease severity, and enable the monitoring of treatment success. In Graves’ disease, natural aAb to the TSH-receptor (TSHR-aAb) are causative for the clinical phenotype, as they bind as endocrine active agonists to the TSH-receptor and stimulate hyperthyroidism and thyroid eye disease [2]. In comparison, natural aAb to another thyroid autoantigen, namely the thyroperoxidase (TPO-aAb), are detectable in both Graves’ disease and Hashimoto’s thyroiditis, where they are associated with cell-mediated cytotoxicity [3]. The TPO-aAb are not directly causing the disease but rather reflect disease activity and disease risk in asymptomatic healthy subjects [4]. Accordingly, TPO-aAb-positive healthy women who become pregnant are, for example, at relatively high risk for the development of postpartum thyroiditis, potentially due to the declining selenium (Se) status during pregnancy [5,6]. The presence of natural aAb in a healthy person without obvious clinical symptoms is neither necessarily an indication of disease nor of immediate diagnostic value but may indicate an increased risk of disease.

In general, the pathogenesis of AITD is a multifaceted process, where the essential production of hydrogen peroxide needed for thyroid hormone biosynthesis may contribute to tissue inflammation, gland destruction, and the generation of novel autoantigens [7,8]. Accordingly, anti-inflammatory and antioxidative measures are considered in prevention and treatment of AITD [9,10]. Observational studies have indicated an inverse relationship between the intake of the essential trace element Se with thyroid volume [11], the development of thyroid nodules [12] and thyroid disease [13]. Dietary uptake of Se is needed for the formation of the 21st proteinogenic amino acid selenocysteine [14], and supports the biosynthesis of health-relevant, redox-active selenoproteins, including the secreted plasma proteins extracellular glutathione peroxidase (GPX3) and selenoprotein P (SELENOP) [15,16,17]. These two selenoproteins are found both in the circulation and in thyroid follicles [18]. While GPX3 is capable of peroxide degradation, SELENOP serves mainly as transporter for the systemic distribution of Se [19,20]. Importantly, renal GPX3 biosynthesis is supported by SELENOP from hepatocytes [21,22].

SELENOP is a most exceptional protein that varies naturally in its primary sequence [23,24]. Translational errors occur during decoding of the ten UGA triplets within the open reading frame [25], and cysteine can replace selenocysteine during biosynthesis [26]. Under antibiotic treatment, Se-free selenoproteins can be synthesized [27]; additionally, tryptophan or arginine may become inserted at UGA codons [28]. Consequently, the average content of Se per SELENOP molecule has been determined at 5.4 ± 0.5 Se/SELENOP in human [29], 7.5 Se/Selenop in rat, and 5 Se/Selenop in mouse [30]. Due to this flexibility in the decoding of UGA, the primary sequence of newly synthesized SELENOP varies. Circulating SELENOP may thus constitute a mixture of slightly different protein variants. For this reason, we hypothesized that autoantibodies to SELENOP (SELENOP-aAb) may develop naturally, particularly in patients with AITD and thyroid inflammation. In order to test this hypothesis, we analyzed control subjects and patients with different thyroid diseases for SELENOP-aAb and correlated the results to biomarkers of Se status.

## 2. Results

**Figure 1:**
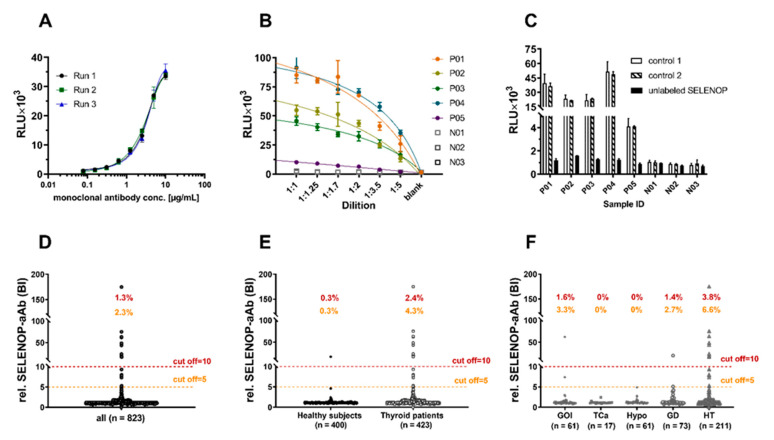
Characterization of the novel autoantibody assay and prevalence of SELENOP-aAb in thyroid patients. (**A**) The SELENOP-aAb assay is characterized by linear response to a SELENOP-specific antibody in gradual dilutions. Signal intensity (RLU—relative light units) correlated to antibody concentration over a range from 0.078–5.0 µg/mL, yielding an R squared by sigmoidal 4 PL curve fitting above 0.99. (**B**) High signal intensities (RLU) decreased gradually with linear dilutions of SELENOP-aAb-positive (P01–P05) samples, but not of negative (N01–N03) samples. (**C**) Suppression of SELENOP-aAb signals by competition with unlabeled SELENOP (1 mg/mL) using equal volumes of sample and unlabeled SELENOP without (control 1) or with BSA (1 mg/mL; control 2). (**D**) Analysis of the full cohort of samples (*n* = 823) yielded a skewed distribution of SELENOP-aAb signals; the thresholds of BI = 5.0 or BI = 10.0 are indicated by dashed lines. (**E**) SELENOP-aAb-positive samples were more prevalent in thyroid patients than in controls (BI > 5; 4.3 vs. 0.3%, or BI > 10; 2.4 vs. 0.3%). (**F**) Among patients, SELENOP-aAb were most prevalent in Hashimoto’s thyroiditis (HT). GOI—goitre; TCa—thyroid carcinoma; Hypo—hypothyroidism; GD—Graves’ disease.

An immunoluminometric assay for the detection and quantification of SELENOP-aAb was established by generating a fusion protein encoding-secreted alkaline phosphatase (SEAP) in frame with full length human SELENOP, where UGA codons had been replaced by cysteine codons. Assay functionality was verified with a SELENOP-specific antibody and showed the expected concentration-dependent signal intensity with dilution (Figure 1A). This result was replicated in dilution experiments with serum samples from patients identified as positive (P01–P05), whereas samples categorized as negative for SELENOP-aAb (N01–N03) showed background signals only (Figure 1B). Unlabeled recombinant SELENOP (1 mg/mL) applied to BSA-free (control 1), or BSA-containing reaction buffer (control 2), was capable of suppressing SELENOP-aAb signals from positive samples, highlighting the specificity of the detection method (Figure 1C).

In order to test for natural SELENOP-aAb in human subjects, serum samples from 2 cohorts of thyroid patients (*n* = 423), along with a collection of healthy controls (*n* = 400), were compared. The results showed a skewed distribution of signals (Figure 1D), and relative binding indices (BI) were calculated by dividing the individual SELENOP-aAb signals by the average signal obtained from the bottom half of all samples. In this first analysis, the thresholds of BI = 5 and BI = 10, respectively, were chosen for the classification of positive vs. negative samples. Irrespective of threshold, SELENOP-aAb were more prevalent in thyroid patients than controls (4.3 vs. 0.3%, or 2.4 vs. 0.3%, respectively) (Figure 1E). Among the patients, elevated SELENOP-aAb was mainly found in patients with Hashimoto’s thyroiditis (Figure 1F).

**Figure 2:**
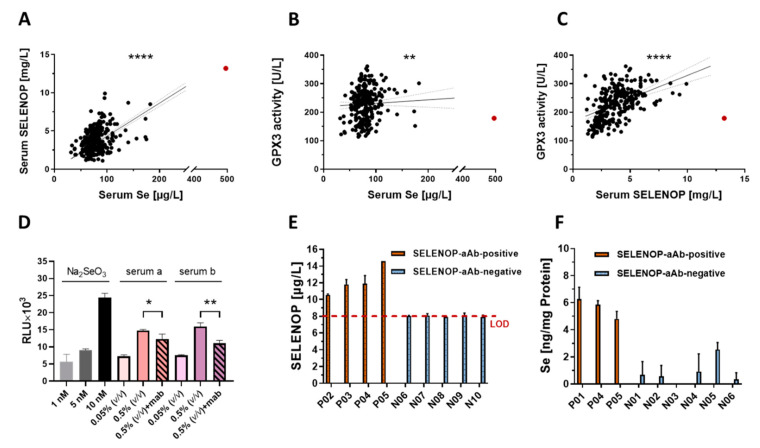
Selenium (Se) status assessment and potential role of natural SELENOP-aAb. Three biomarkers of Se status were determined in serum samples from AITD patients (*n* = 284). Positive correlations were observed for (**A**) total Se with SELENOP (r = 0.342, *p* < 0.0001), (**B**) total Se with GPX3 activity (r = 0.171, *p* = 0.0046), and (**C**) SELENOP with GPX3 activity (r = 0.545, *p* < 0.0001). The sample with highest SELENOP-aAb displayed exceptionally high Se and SELENOP levels in combination with moderate GPX3 activity (indicated as red dots). (**D**) HEK293 cells expressing a Se-dependent luciferase showed increased reporter activity (RLU) in response to selenite or human serum added to the culture medium. The signal was significantly suppressed by the addition of a SELENOP-specific antibody (*n* = 3). (**E**) The immunoglobulins from 5 SELENOP-aAb-positive (P01–P05) and 10 SELENOP-aAb-negative samples (N01–N10) were precipitated by protein A. Measurable (**E**) SELENOP or (**F**) Se concentrations were detected in the isolates from SELENOP-aAb-positive samples only, but not in those from SELENOP-aAb-negative samples. Correlations were analyzed by Spearman’s correlation test. Two-tailed *t*-test was used for comparisons between two groups, *p*-values < 0.05 were considered statistically significant; * indicates *p* < 0.05; ** indicates *p* < 0.01; **** indicates *p* < 0.0001.

Next, the impact of SELENOP-aAb on the Se status was analyzed in the AITD patients. Three complementary biomarkers of Se status, i.e., total serum Se and SELENOP concentrations along with GPX3 activity, were assessed in parallel. Serum SELENOP showed positive and strong linear correlations with total Se and GPX3 activity (Figure 2A,C), while total serum Se correlated only weakly with GPX3 activity (Figure 2B). One particular sample displayed moderate GPX3 activity in combination with exceptionally high Se and SELENOP concentrations (indicated as red dot; Se—495 µg/L; SELENOP—13.2 mg/L; GPX3 activity—179 U/L). This finding may indicate a compromised *GPX3* gene expression, a mutated GPX3 variant with reduced enzymatic activity, accelerated GPX3 degradation, or little renal GPX3 biosynthesis, potentially due to impaired renal Se uptake. This sample displayed highest SELENOP-aAb concentrations (BI = 175), in agreement with the hypothesis of a disturbed Se transport towards the kidney. In order to test whether antibodies to SELENOP are capable of affecting target cell Se uptake, human embryonic kidney cells (HEK293) were transfected with a reporter for selenoprotein biosynthesis, encoding a Se-dependent luciferase in combination with the selenocysteine insertion sequence (SECIS) element of GPX4. The reporter system showed Se-dependent induction of luciferase activity in response to inorganic selenite (1.0, 5.0 or 10 nM, f.c.), and in response to human serum (0.05% or 0.5%, *v*/*v*) as a natural Se source, as expected under regular Se-deficient culture conditions with 10% FBS as sole Se source. A significant decline in the luciferase reporter signal was observed upon adding an antibody to SELENOP (0.8 ng/mL), into the culture medium (Figure 2D). This result is compatible with the notion of SELENOP-aAb disturbing SELENOP uptake into target cells. To further verify the characteristics of natural SELENOP-aAb, immunoglobulins were isolated from positive and negative serum samples by protein A-mediated immunoprecipitation and analyzed for directly associated Se and SELENOP content. The isolates from SELENOP-aAb-positive samples contained measurable amounts of Se (Figure 2E) and/or SELENOP (Figure 2F), whereas isolates from controls were devoid of associated Se or SELENOP.

**Figure 3:**
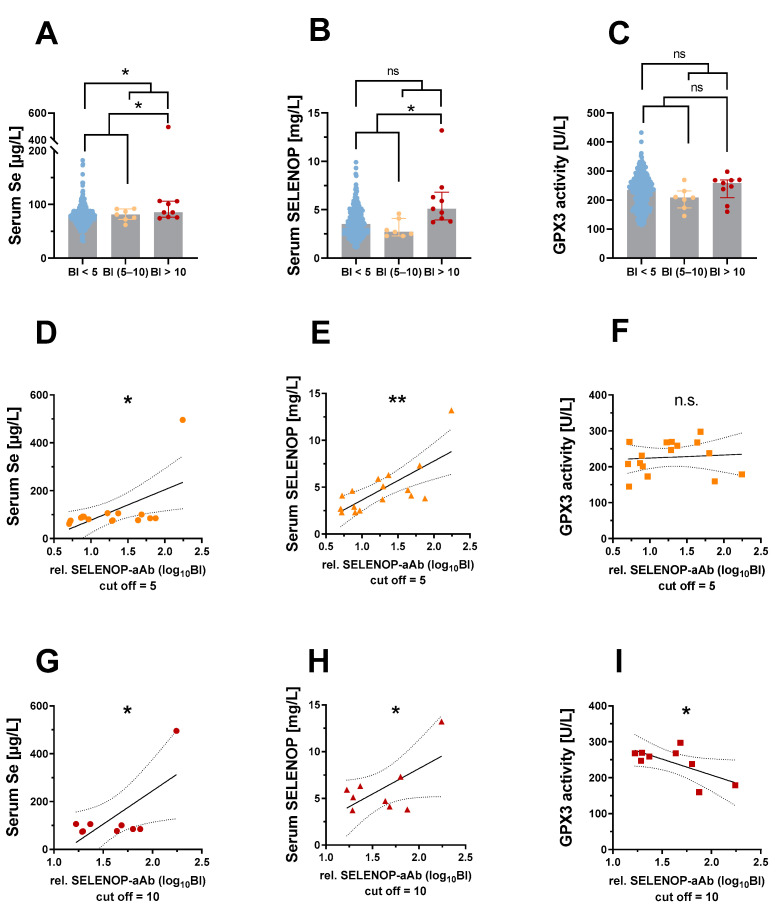
Comparison of three complementary biomarkers of Se-status in relation to SELENOP autoimmunity. The patients with positive SELENOP-aAb (BI > 5) displayed relatively high (**A**) total Se, while (**B**) SELENOP or (**C**) GPX3 activity were not different in comparison to SELENOP-aAb-negative patients. SELENOP-aAb correlated positively to (**D**) serum Se and (**E**) SELENOP, but not to (**F**) GPX3 activity. When applying a more stringent cut-off for positivity (BI > 10), serum samples with positive SELENOP-aAb displayed relatively high (**A**) total Se and (**B**) SELENOP, whereas (**C**) GPX3 activity was not elevated. Positive correlation was observed between SELENOP-aAb and (**G**) serum Se and (**H**) SELENOP. (**I**) GPX3 activity was inversely correlated to SELENOP-aAb. Comparisons between two groups were conducted by Mann–Whitney test. Correlations were tested by Pearson’s correlation analysis with two-tailed *p*-values. *p*-values < 0.05 were considered statistically significant; n.s. indicates *p* ≥ 0.05; * indicates *p* < 0.05, and ** indicates *p* < 0.01.

When classifying patients as SELENOP-aAb-positive or -negative, by choosing BI > 5.0 as threshold, the positive samples displayed elevated total serum Se (median: 85.3 vs. 77.0 µg/L, *p* = 0.033), whereas SELENOP concentration or GPX3 activity was not different (Figure 3A–C). A positive correlation was observed between SELENOP-aAb with Se (r = 0.582, *p* = 0.018) (Figure 3D) and with SELENOP (r = 0.730, *p* = 0.001) (Figure 3E), but not with GPX3 activity (Figure 3F). When using a higher threshold, i.e., BI > 10 as cut-off, relatively high Se (85.3 vs. 77.1 µg/L, *p* = 0.0178) and SELENOP (5.1 vs. 3.5 mg/L, *p* = 0.001) concentrations were observed in the group of SELENOP-aAb-positive samples (Figure 3A,B), whereas GPX3 activity was not elevated (Figure 3C). Positive correlation was observed between SELENOP-aAb and Se (r = 0.695, *p* = 0.038) (Figure 3G) or SELENOP (r = 0.627, *p* = 0.035) (Figure 3H), reaching statistical significance when the very positive sample highlighted in Figure 2A–C is included, but not when omitted from the analysis. Notably, the SELENOP-aAb correlated inversely to GPX3 activity (r = −0.669, *p* = 0.049) (Figure 3I).

## 3. Discussion

This study describes naturally occurring autoantibodies to the Se transport protein SELENOP in human subjects and suggests a potential physiological relevance. Our data indicate that autoimmunity to SELENOP is a rare finding in healthy adult subjects. However, a considerable fraction of thyroid patients express SELENOP-aAb to varying degrees, with some patients being highly positive for SELENOP-aAb. The elevated prevalence in Hashimoto’s thyroiditis may either result from SELENOP-aAb predisposing them to the diseases—as low Se has been identified as risk factor for autoimmune thyroid disease [31,32,33]—or the autoimmunity develops as a consequence of the ongoing inflammation and associated oxidative damage to thyroid proteins, i.e., resulting from the lymphocytic thyroiditis and generation of novel autoantigens as side effect of disease [8,34]. Alternatively, both the predisposition to and consequences of the inflammatory disease may have contributed to the elevated prevalence of SELENOP-aAb observed in the patients [6]. From an etiological perspective, human SELENOP is naturally synthesized in different variants, some of which are potentially prone to becoming easily modified and eliciting an autoimmune response, as the protein carries several highly reactive selenocysteine residues [35,36]. Both notions may offer an explanation for the increased prevalence of SELENOP-aAb in AITD and indicate a diagnostic or even pathophysiological relevance, as just recently documented for the central role of Se status and GPX-dependent protection from neutrophil ferroptosis in systemic autoimmunity [37]. This hypothesis needs to be elucidated in larger studies and ideally with samples from a longitudinal, prospective trial.

Against our expectation, SELENOP-aAb were not associated with SELENOP deficiency, but rather with elevated Se and SELENOP serum concentrations, suggesting some stabilizing effect of the autoantibodies on circulating SELENOP. This interpretation is consistent with the observed inhibition of SELENOP uptake into renal HEK293 target cells by antibodies to SELENOP, nicely echoing published results on SELENOP-neutralizing antibodies antagonizing Se uptake as novel candidates for type 2 diabetes therapy [38,39]. Importantly, this theory is substantiated in human subjects by the relatively low and inadequate GPX3 activity in SELENOP-aAb-positive patients, and the inverse association of GPX3 activity with SELENOP-aAb concentrations, despite an increased Se status as reflected in elevated total Se and SELENOP levels. Transgenic mouse models have shown that hepatic SELENOP biosynthesis is supplying the kidney with Se for GPX3 biosynthesis via specific uptake through the lipoprotein receptor megalin [22,40]. In the case that the antagonistic nature of SELENOP-aAb, impairing Se supply to target cells, becomes substantiated by future research, it would be of far-reaching medical relevance, as also the central nervous system relies on receptor-mediated SELENOP uptake for Se supply and protection of highly active interneurons from death by ferroptosis [41,42]. In the absence of regular and efficient SELENOP supply and uptake, severe neurological symptoms, including epileptic seizures, were observed in transgenic mice [41,43,44,45,46], and also recently in a dog model of impaired SELENOP expression, leading to brain atrophy and cerebellar ataxia [47]. It remains to be tested whether SELENOP-aAb are relevant for neurological disease, and whether SELENOP-aAb-positive thyroid patients are at particular risk for neurological sequelae, e.g., seizures, tremors, ataxia, or symptoms of Hashimoto encephalopathy.

Correcting a Se deficit, whatever the cause, needs an adequate supply, as excessive Se intake can be toxic [48]. Some observational studies and systematic analyses have reported an association between elevated Se concentrations and type 2 diabetes [49]. Other systematic reviews, focusing on sufficiently large and well-controlled supplementation studies, show that there is no evidence that Se supplementation increases the risk of type 2 diabetes [50]. The documented association between increased serum Se concentrations and type 2 diabetes could be a consequence of insulin resistance leading to increased hepatic biosynthesis of SELENOP due to abrogation of insulin-mediated SELENOP suppression, i.e., a question of reverse causality, potentially a function of protecting the cardiovascular system [51]. This notion is supported by recent findings of a positive association between increased Se concentrations and protection from all-cause mortality and heart disease mortality in diabetic patients, as determined in NHANES III [52]. The extent to which SELENOP-aAb may alter the protective interaction of Se and SELENOP on the vasculature is unknown, and the question of whether functional Se deficiency by low Se supply, in combination with SELENOP-aAb causes hypoglycemia needs to be investigated [53].

**Figure 4:**
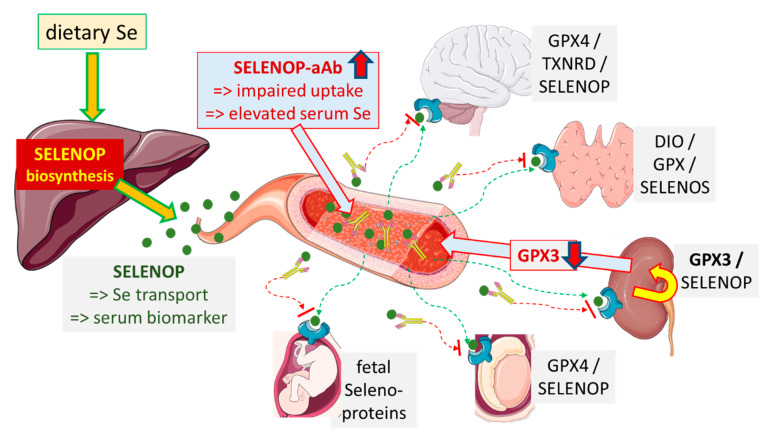
Potential pathophysiological relevance of SELENOP-aAb. Dietary sources of Se are mainly converted in hepatocytes into SELENOP for secretion and systemic supply of target tissues. Thereby, SELENOP constitutes both the major serum Se transporter and a reliable biomarker of Se status. Patients with elevated SELENOP-aAb display increased serum Se and SELENOP concentrations, indicative of SELENOP stabilization or impaired receptor-mediated SELENOP uptake and clearance. Thereby, target tissues may become insufficiently supplied for full biosynthesis of the essential tissue-relevant selenoproteins (examples are indicated). The biosynthesis of circulating GPX3 by kidney cells is known to directly depend on hepatic SELENOP. Accordingly, serum GPX3 activity correlated inversely to SELENOP-aAb in thyroid patients, consistent with target cell Se deficiency. Extrapolating these results, SELENOP-aAb may increase the risk of neurological symptoms, thyroid disease, subfertility, pregnancy problems, and other Se-dependent diseases.

Our findings suggest that Se status assessment by the biomarkers used until now, i.e., total Se and SELENOP concentrations along with GPX3 activity [54,55,56,57,58], may be insufficient when SELENOP-aAb are present. In particular, the poor correlation between Se or SELENOP concentrations and GPX3 activity in a given subject may indicate the presence of SELENOP-aAb impairing SELENOP uptake and GPX3 biosynthesis by kidney, as impressively documented in the positive subject with highest SELENOP-aAb (red dots in Figure 2). This interrelation may also be of relevance for preventive or adjuvant Se therapy [33,59,60,61,62], as positive effects may be observed, particularly in the presence of SELENOP-aAb. This hypothesis is based on the findings in transgenic mice, where SELENOP deficiency and resulting symptoms can be successfully compensated for by supplemental selenite or other selenocompounds [45,63,64,65]. A score combining classical Se status biomarkers with the presence of SELENOP-aAb may be superior in judging the functional Se status and requirements for Se supplementation than a single biomarker alone. Such a scoring system would be capable of identifying Se deficiency also in cohorts of well-supplied subjects with apparently sufficient SELENOP expression, and might indicate subjects with specific requirements to overcome SELENOP-aAb-mediated inhibition of SELENOP-dependent Se supply (Figure 4). Prospective studies of sufficient size and length are needed next to test the clinical relevance of SELENOP-aAb for AITD risk, and for stratifying the results from Se supplementation studies.

## 4. Materials and Methods

### 4.1. Human Samples

Blood samples (*n* = 423) from patients with different thyroid diseases were collected and serum was prepared, aliquoted, and stored at −80 °C. The study had been approved by the Ethical committee of the Charité-University Medical School, Berlin (#EA2/173/17). Recruitment of the patients proceeded in consecutive manner and diagnostic criteria for thyroid disease were applied as described in [66]. A cohort of serum samples from subjects with a self-reported status as healthy (controls, *n* = 400) was obtained from a commercial supplier (InVent Diagnostica GmbH, Hennigsdorf, Germany) and served as control (Table 1). All samples included were derived after obtaining written informed consent from the subjects enrolled into the analyses, and the study was conducted in accordance with the declaration of Helsinki on ethical principles for medical research involving human subjects.

### 4.2. Generation of Recombinant SEAP-SELENOP Reporter Proteins

The cDNA of secreted alkaline phosphatase (SEAP) was amplified by PCR and inserted into plasmid pIRESneo (Clontech, Palo Alto, CA, USA) giving rise to pIRESneo-SEAP. A cDNA of a UGA-free human SELENOP reading frame was synthesized, whereby UGA codons were replaced by cysteine codons by a commercial supplier (Eurofins Genomics GmbH, Ebersberg, Germany). The fragment was amplified by PCR and ligated into pIRESneo-SEAP, amplified in E. coli and sequence verified. Recombinant protein was produced via stable expression of pIRESneo-SEAP-SELENOP in HEK 293 cells. Cell culture supernatants containing the secreted recombinant protein were collected and used as reporter in the SELENOP-aAb detection assay.

### 4.3. Immunoluminometric Assay for Detection of SELENOP-aAb

The immunoluminometric assay is based on the binding of aAb to recombinant SEAP-SELENOP, followed by precipitation of the antibody–antigen–reporter complex by protein A. To this end, diluted supernatants of HEK293 cells expressing SEAP-SELENOP was incubated with 5 μL serum sample at 4 °C overnight. On the second day, the same volume (40 µL) of a protein A slurry (ASKA Biotech GmbH, Berlin, Germany) was added and incubated for 1 h at RT. Complexes formed were washed 6 times with washing buffer (50 mM Tris HCl, pH 7.4, 100 mM NaCl, 10% glycerol, and 0.5% Triton X-100), removing unbound SEAP-SELENOP and other serum proteins. Finally, reporter activity was detected as luminescence signal by a luminometer (Berthold Technologies GmbH, Bad Wildbad, Germany). Binding index (BI) was calculated for each sample by dividing the relative light units (RLU), obtained with the average RLU from the lowest 50% of signals in the same assay plate, which was set as BI = 1.0. This mathematical method for background signal definition was based on the assumption that SELENOP-aAb were present in less than 50% of the samples tested. During the analyses, the inter- and intra-assay CV was determined to be below 20% based on the analysis of selected positive and negative serum samples included into each assay run.

### 4.4. Isolation of Immunoglobulins (IgG) from Serum Sample

Total IgGs were isolated from five positive and ten negative serum samples by precipitation with Protein A slurry (ASKA Biotech GmbH, Berlin, Germany). To this end, serum samples were incubated with 2 volumes of protein A in PBS (50%) overnight at 4 °C. The supernatants were discarded, and the pellets were washed 6 times with PBS. Precipitated IgG were eluted with 3-fold volume of citric acid (25 mM, pH 2.0) and neutralized using 1-fold volume of HEPES (1 M, pH 8.0).

### 4.5. Quantification of GPX3 Activity and Total Se and SELENOP Concentrations

GPX3 activity in serum samples was determined by a coupled enzymatic test monitoring NADPH decline at 340 nm due to glutathione reductase activity catalyzing regeneration of consumed glutathione by GPX during H_2_O_2_ reduction [67]. Briefly, serum samples of 5 µL were applied to 96-well plates containing 200 µL of a test mixture, including 1 mM NaN_3_, 3.4 mM reduced glutathione, 0.3 U/mL glutathione reductase, and 0.27 mg/mL NADPH. The reaction was started by 10 µL of 0.00375% H_2_O_2_. A constant serum sample was included into each assay run for quality control. The inter- and intra-assay CVs were determined to be below 15% during the analyses.

Se concentrations in serum samples or in the immunoglobulin-isolates were analyzed by total reflection X-ray fluorescence (TXRF) using a benchtop TXRF analyzer (S4 T-STAR, Bruker Nano GmbH, Berlin, Germany), as described previously [68,69]. Briefly, serum samples were diluted with a Ga-standard (1 mg/L, Alfa Aesar GmbH & Co KG, Karlsruhe, Germany), applied to polished quartz glass plates, and dried at 37 °C overnight. Isolated immunoglobulin complexes from SELENOP-aAb-positive and -negative serum samples were precipitated by adding 9 volumes of ice-cold ethanol (100%) overnight at −80 °C, and subsequent centrifugation for 30 min at 4 °C and 14,000× *g*. The pellets were dissolved at 80 °C for 2 h in 100 µL HNO_3_ (61%), the Ga standard was added, and sample was applied to quartz plates and analyzed. A commercial serum standard (Seronorm, Sero AS, Billingstad, Norway) served as control in each analytical run. The determined concentrations of Se were within the specified range of the standard, and the inter-assay coefficient of variation (CV) was below 5% during the analyses.

SELENOP concentrations were determined by a validated commercial SELENOP-specific ELISA (selenOtest ELISA, selenOmed GmbH, Berlin, Germany), essentially as described in [29]. Briefly, 100 µL of each IgG-isolate or 1:33 diluted serum samples were applied to pre-coated 96-well plates. Standards and calibrators were included into each assay run for quality control. The inter-assay CV was determined to be below 10%.

### 4.6. Luciferase-Based Reporter Gene Assay for Selenoprotein Biosynthesis in Cell Culture

A Se-responsive reporter gene assay was established using stably transfected human embryonic kidney (HEK293) cells, expressing a luciferase reporter containing a fusion protein of Firefly (FLuc) and Renilla (RLuc) luciferase, interrupted by an in-frame UGA codon and a GPX4-derived selenocysteine-insertion sequence (SECIS) element, as described previously [70]. Cells were cultured in Dulbecco’s Modified Eagle Medium (DMEM/F12; Pan-Biotech GmbH, Aidenbach, Germany), containing 10% (*v*/*v*) FCS. For reporter gene assay, 20,000 cells per well were cultivated in 96-well plates in DMEM/F12 containing 10% (*v*/*v*) FCS for 24 h and stimulated by different sources of Se (sodium selenite or human serum) in the absence or presence of an anti-SELENOP antibody (0.8 ng/mL f.c., #SM-MAB-7356, selenOmed GmbH, Berlin, Germany).

The two human sera used in this experiment were tested free of SELENOP-aAb. The Se concentrations of these serum samples used in the in vitro studies were (a) 100.6 µg/L and (b) 109.7 µg/L, corresponding to the final Se concentrations in the cell culture mediums (0.5% serum, *v*/*v*), of 6.4 nM and 6.9 nM, respectively. After 48 h of incubation, medium was removed and cells were lysed in 40 µL passive Lysis puffer (PromoCell, Heidelberg, Germany) for 10 min at RT and stored at −30 °C to support cell lysis until measurement. For the detection, 25 µL of cell lysates were transferred into white 96-well plates, and Renilla luciferase activity was measured 30 s after adding coelenterazine (100 µL/well, 2.5 µg/mL in PBS, Synchem UG, Altenburg, Germany) using a microplate reader (PerkinElmer, Waltham, MA, USA).

### 4.7. Statistical Analyses

Statistical analyses were performed using SPSS (version 25, SAS Institute, Cary, NC, USA) or GraphPad Prism v.9.1.2 (GraphPad Software Inc., San Diego, CA, USA). The results are represented as mean with SD, as median with interquartile range, or by displaying the individual values. Normal distribution of values was tested by the Shapiro–Wilk test. Comparisons between two groups were conducted by unpaired *t*-test, and non-normally distributed variables were compared with the Mann–Whitney test. Correlations were tested by Pearson’s correlation analysis and for non-normally distributed variables by Spearman’s correlation test. *p*-values < 0.05 were considered significant; * *p* < 0.05, ** *p* < 0.01, *** *p* < 0.001, **** *p* < 0.0001.

## 5. Conclusions

The results indicated an impairment of SELENOP-dependent Se transport by natural SELENOP autoantibodies, and suggest that the characterization of health risk from Se deficiency may need to include autoimmunity to SELENOP as additional biomarker of Se status. Thereby, patients with “functional Se deficits” may be identified as those who may display a particular requirement for a sufficiently high Se intake and are likely to respond positively to adjuvant Se in clinical supplementation trials.

## Figures and Tables

**Table 1:** Characterization of the study cohort.

Healthy Controls	*n* = 400
sex, female/male [*n*/*n*]	200/200
age, median (95% CI) [y]	31 (29–32)
Thyroid Patients	*n* = 423
sex, female/male [*n*/*n*]	362/61
age, median (95% CI) [y]	49 (47–51)
GOI, *n* (%)	61 (14.4%)
TCa, *n* (%)	17 (4.0%)
Hypo, *n* (%)	61 (14.4%)
GD, *n* (%)	73 (17.3%)
HT, *n* (%)	211 (49.9%)

GOI—goitre; TCa—thyroid carcinoma; Hypo—hypothyroidism; GD—Graves’ disease; HT—Hashimoto’s thyroiditis.

## Data Availability

The data presented in this study are available upon reasonable request from the corresponding author.
